# Metal-Free Doping Strategies in Two-Dimensional Carbon Nitride C_4_N_2_ for Enhanced Hydrogen Evolution Catalysis

**DOI:** 10.1021/acsomega.5c05773

**Published:** 2025-09-16

**Authors:** Bruno Ipaves, João F. Justo, James M. de Almeida, Lucy V. C. Assali, Pedro Alves da Silva Autreto

**Affiliations:** † Center of Natural and Human Sciences, 425753 Federal University of ABC (UFABC), Santo André, 09280-560, São Paulo, Brazil; ‡ Escola Politécnica, University of São Paulo (USP), São Paulo 05508-010, São Paulo, Brazil; § Ilum School of Science, 67420 Brazilian Center for Research in Energy and Materials (CNPEM), Campinas, 13083-970 São Paulo, Brazil; ∥ Institute of Physics, University of São Paulo (USP), São Paulo 05508-090, São Paulo, Brazil

## Abstract

This study investigates the structural, electronic, and catalytic properties of pristine and doped C_4_N_2_ nanosheets as potential catalysts for the hydrogen evolution reaction (HER). The pristine C_36_N_18_ nanosheets exhibit limited HER activity, primarily due to high positive Gibbs free energies (>2.2 eV). We explored doping it with B, Si, or P atoms at the nitrogen site to enhance catalytic performance. Among these systems, B-doped C_36_N_17_ nanosheets exhibit the most promising catalytic activity, with a Gibbs free energy close to zero (≈−0.2 eV), indicating efficient hydrogen adsorption. Band structure, projected density of states (PDOS), charge density, and Bader charge analyses reveal significant changes in the electronic environment due to doping. Although stacking configurations (AA′A^″^ and ABC) have a minimal effect on catalytic performance, doping, particularly with B, substantially alters the electronic structure, thus optimizing hydrogen adsorption and facilitating an efficient hydrogen evolution reaction.

## Graphical abstract

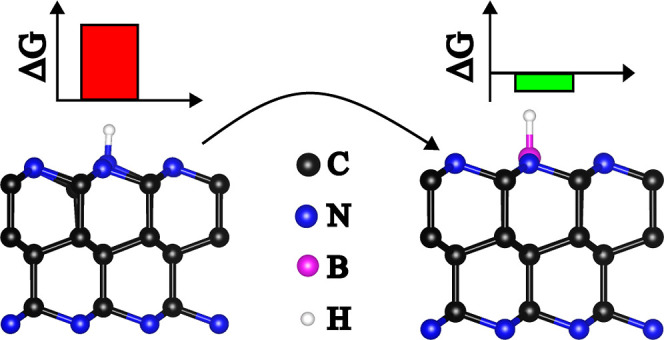

## Introduction

Given the increasing global energy demand and environmental crisis, developing sustainable energy systems to produce clean fuels and chemicals has gained increasing interest. These systems are essential to replace fossil fuels and mitigate carbon dioxide emissions, which are major contributors to climate change.^1,2^ Among clean energy carriers, hydrogen (H_2_) stands out due to a unique set of characteristics, such as nontoxicity, renewability, zero pollution, high abundance, and the highest energy density per unit mass, making it a promising candidate for a primary energy source.^2,3^

Nevertheless, the widespread adoption of hydrogen as a sustainable energy carrier depends on its production from clean and renewable sources, often referred to as “green hydrogen.” Green hydrogen, generated through processes such as water electrolysis powered by renewable energy, has the potential to contribute significantly to the decarbonization of energy systems.^4,5^ In addition, the ability of hydrogen to store energy efficiently positions it as a key solution for large-scale energy storage, addressing the problem of intermittent renewable energy sources, such as solar and wind. This dual role—as both a clean fuel and a medium for energy storage at the grid level—underscores its importance in the transition toward carbon-neutral power systems.^4,5^ Furthermore, the development of metal-free catalysts has emerged as a strategic approach to promote sustainable hydrogen production by reducing costs and avoiding the use of critical metals, which is essential to enable large-scale clean energy technologies.^6,7^

In this context, developing efficient electrocatalysts for the hydrogen evolution reaction (HER) is critical to enable green hydrogen production through water electrolysis. Among the materials investigated, two-dimensional (2D) systems have garnered significant attention since the isolation of graphene, due to their unique properties.^2,8^ These materials provide large surface areas, tunable electronic properties, and chemical versatility, making them ideal for several applications, including batteries,^9^ sensors,^10,11^ oxygen reduction reaction (ORR),^12^ and HER.^8,13^ Consequently, the investigation of 2D materials and the exploration of novel strategies, such as doping or defect engineering, to enhance their catalytic properties, has become a key focus in the search for sustainable energy solutions.

Among 2D materials, carbon nitride-based nanosheets have shown considerable promise in enhancing HER efficiency by providing abundant active sites for hydrogen adsorption and improving reaction kinetics.^2,3,8,13^ Recently, C_4_X_2_ (X = B or N) nanosheets with AA′A^″^ and ABC stacking have been explored through first-principles calculations, revealing their intriguing structural and electronic properties.^14^ However, their potential catalytic activity for HER remains unexplored, leaving a gap in understanding their suitability as catalysts for sustainable hydrogen production.

In the present work, we investigate the structural, electronic, and catalytic properties of pristine C_4_N_2_ nanosheets in AA′A^″^ and ABC stacking using first-principles calculations, along with boron (B), silicon (Si), and phosphorus (P)-doped systems. Our study evaluates the Gibbs free energy of hydrogen adsorption, electronic structure modifications, and the role of doping in optimizing HER performance, providing insight into the potential of C_4_N_2_ nanosheets as efficient catalysts.

## Computational Details

This study used first-principles calculations based on spin-polarized Density Functional Theory (DFT),^15,16^ utilizing the plane-wave basis set and projector augmented-wave (PAW) method,^17^ as implemented in the Quantum ESPRESSO software package.^18,19^ The generalized gradient approximation of Perdew–Burke–Ernzerhof (GGA-PBE) exchange–correlation functional^20^ was applied, along with the Dion et al. scheme^21^ refined by Klimeš et al. (optB88-vdW^22^) to accurately account for van der Waals (vdW) interactions. The plane-wave energy cutoff was set at 80 Ry, with a total energy convergence threshold of 0.1 meV/atom. To sample the irreducible Brillouin zone^23^ a 6 × 6 × 1 k-point mesh was utilized.

We constructed hexagonal supercells based on the primitive cell of the 2D structures previously investigated.^14^ The cell parameters in the *xy*-plane were determined through variable cell optimization using the BFGS quasi-Newton algorithm. To avoid interactions between cell images, we fixed the lattice parameter perpendicular to the sheets (along the *z*-axis) at 25 Å. This approach has proven effective in analogous 2D systems in prior studies.^24–26^ To investigate the interaction between the H atom and the doped C_36_N_17_, we initially placed H at approximately 1 Å from the dopant.

We investigated the HER activities of pristine and doped nanosheets using the Sabatier principle.^3^ According to this principle, the catalyst must bind strongly to the intermediate to facilitate the reaction. In contrast, the product (hydrogen atoms) should be weakly bound to the surface to allow for rapid desorption. At equilibrium, the efficiency of HER catalysis on a surface is dictated by the exchange current density, which is related to the Gibbs free energy Δ*G* under standard conditions (acidic medium, pH → 0), and can be defined as follows^3^

1 $$\Delta G=\Delta E_{ads}+E_{ZPE}-T\Delta S$$

where Δ*E*_ads_ is the adsorption energy of a hydrogen (H) atom, *E*_ZPE_ is the change in zero-point energy and *T*Δ*S* is the entropy change between the adsorbed and gaseous states at 298.15 K. The hydrogen adsorption energy is given by^3^

2 $$\Delta E_{ads}=E_{\left(System+H\right)}-E_{\left(System\right)}-\frac{1}{2}E_{\left(H_{2}\right)}$$

where *E*_(System+H)_ is the total energy of hydrogen adsorbed on pristine or doped systems, *E*_(System)_ is the energy of pristine or doped systems without H adsorption, and $E_{\left(H_{2}\right)}$ is the energy of an isolated hydrogen molecule. Given that the *E*_ZPE_ and *T*Δ*S* contributions are nearly identical across the different surfaces, eq 1 can be approximated by^3,27^

3 $$\Delta G=\Delta E_{ads}+0.24eV$$

The work function of our systems was determined using the expression

4 $$\Phi =V_{\infty }-E_{F}$$

where Φ is the work function, *E*_F_ is the Fermi energy, and *V*_∞_ is the vacuum level or electrostatic potential. For semiconductor systems, *E*_F_ = (*E*_VBM_ + *E*_CBM_)/2, where VBM is the valence band maximum and CBM is the conduction band minimum.

We computed the charge density differences for H adsorbed on pristine or doped nanosheets to better understand the charge transfer. These differences were determined using the equation

5 $$\Delta \rho =\rho_{\left(System+H\right)}-\rho_{\left(System\right)}-\rho_{\left(H\right)}$$

where ρ_(System+H)_, ρ_(System)_, and ρ_(H)_ are, respectively, the charge densities of H adsorbed on pristine or doped systems, pristine or doped systems, and isolated H atom. Bader charge analysis was also performed to quantify the charge transfer from the H atom to the C_4_N_2_.^28,29^

## Results and Discussion

We initially optimized a 3 × 3 × 1 supercell of the C_4_N_2_ nanosheets, considering both AA′A^″^ and ABC stacking configurations. The supercell consisted of 54 atoms, including 36 carbon (C) and 18 nitrogen (N) atoms. Since both stacking arrangements exhibited similar structural and electronic properties, we present all figures for the ABC stacking in the Supporting Information. Figures 1 and S1 in the Supporting Information illustrate the configuration of these systems, while Table 1 provides the corresponding structural parameters.

**Figure 1:**
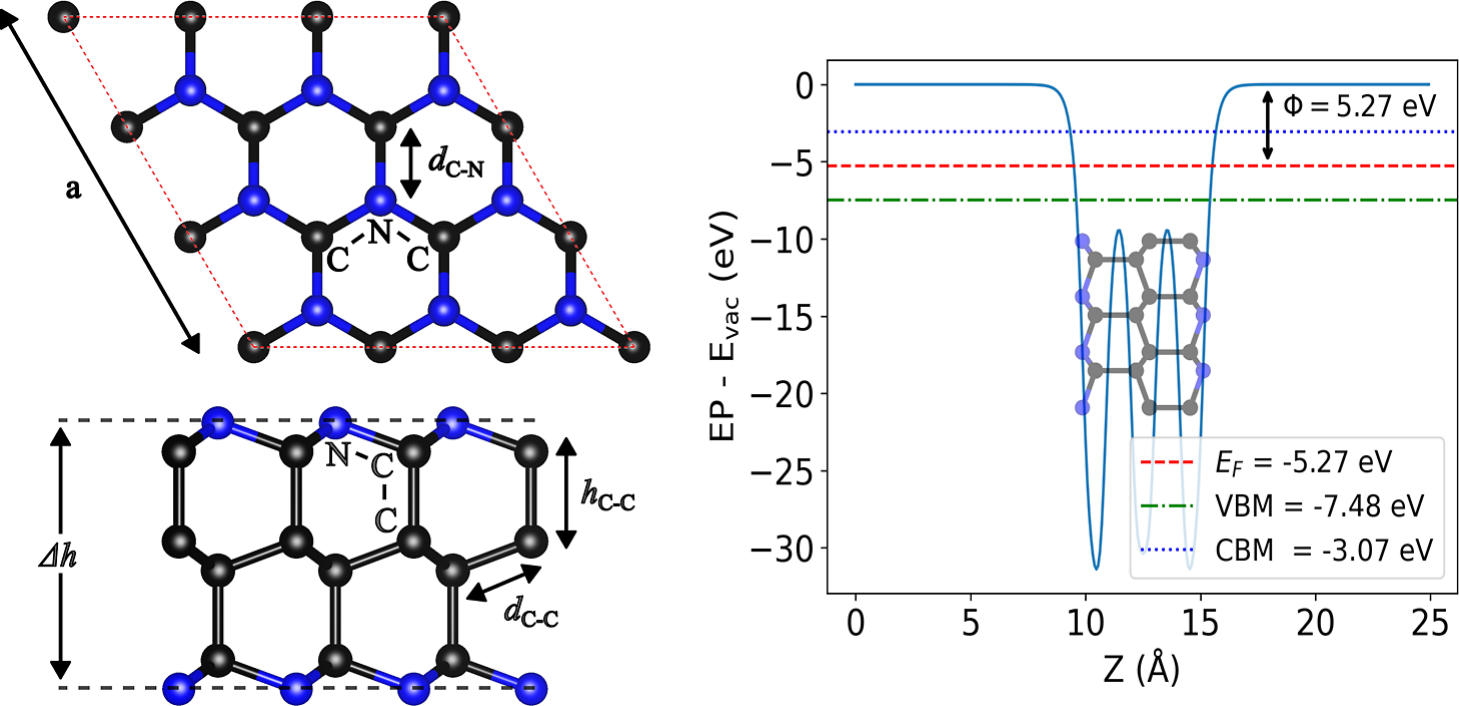
Schematic representation, presented in top and side views (left), and work function Φ (right) of optimized pristine AA′A^″^-C_36_N_18_ nanosheet. Structural parameters (lattice constants, bond lengths, and bond angles) are highlighted. The respective values are given in Table 1. The work function, Fermi energy, VBM, and CBM are referenced relative to the vacuum energy level. The corresponding details for the ABC stacking configuration are provided in Figure S1 in the Supporting Information.

**Table 1:** Structural and Electronic Properties of Pristine and Hydrogen-Adsorbed C_36_N_18_: Lattice Parameter (*a*), Intralayer (*d*) and Interlayer (*h*) Distances, Thickness (Δ*h*), Intraplanar (C–N–C) and Interlayer (C–C–N) Bond Angles, Electronic Band Gap (*E*_g_), Work Function (Φ), and Gibbs Free Energies (Δ*G*), as Labeled in Figures 1 and 2 (AA′A^″^) and S1 and S2 in the Supporting Information (ABC). Distances are Given in Å, Energies in eV, and Angles in Degrees

system	*a*	bond length			Δ*h*	H distance	angle		*E*_*g*_	Φ	Δ*G*
AA′A^″^		*d*_C–C_	*h*_C–C_	*d*_C–N_			C–C–N	C–N–C			
pristine	7.27	1.49	1.60	1.49	4.74		110.07	108.86	4.41	5.27	
H C_Top_	7.27	1.49	1.60	1.49	4.74	3.23 (C–H)	110.07	108.86	4.22		2.59
H hollow	7.27	1.49	1.60	1.49	4.74	3.21 (R–H)	110.07	108.86	4.21		2.59
H N_Top_	7.32	1.50	1.60	2.05	4.83	1.03 (N–H)	112.85	113.80	0.60		2.23
ABC											
pristine	7.30	1.50	1.56	1.49	4.66		109.82	109.12	4.14	5.32	
H C_Top_	7.30	1.50	1.56	1.49	4.66	3.23 (C–H)	109.82	109.12	3.96		2.59
H hollow	7.30	1.50	1.56	1.49	4.66	3.20 (R–H)	109.82	109.12	3.97		2.59
H N_Top_	7.36	1.50	1.56	2.05	4.79	1.03 (N–H)	113.48	113.81	0.78		2.28

The lattice parameters are 7.27 and 8.66 Å for the AA′A^″^ and ABC configurations, respectively. For the AA′A^″^ stacking configuration, the intralayer C–C bond length (*d*_C–C_) and interlayer C–C distance (*h*_C–C_) are 1.49 and 1.60 Å, respectively. The thickness (Δ*h*) is 4.74 Å, and the C–N bond length (*d*_C–N_) is 1.49 Å. In the ABC stacking configuration, the corresponding values are 1.50, 1.56, 4.66, and 1.49 Å, respectively. The C–C–N bond angle is 110.07° for the AA′A^″^ stacking configuration, while the C–N–C bond angle is 108.86°. These angles are, respectively, 109.84° and 109.10° for the ABC stacking configuration.

The work function (ϕ) is frequently considered as a descriptor of catalytic activity in hydrogen evolution reactions, as it reflects the material’s ability to transfer electrons to adsorbed species. However, its exact role remains a subject of debate in the literature. Some studies suggest that a lower ϕ facilitates electron injection and thus enhances HER activity, particularly for semiconducting systems where the position of the Fermi level relative to the vacuum level can influence reaction kinetics.^30^ On the other hand, other studies argue that HER performance is optimized when ϕ approaches that of noble metals such as Pd (5.12 eV) or Pt (5.65 eV), which exhibit ideal binding energies for hydrogen.^30,31^

In this context, our calculated ϕ values for the pristine C_4_N_2_ systems fall within this range (see Figures 1 and S1, Table 1), consistent with experimental reports for carbon-based materials containing specific defect and doping patterns. For instance, Jia et al.^32^ performed local work-function measurements using Kelvin probe force microscopy (KPFM) and demonstrated that defective nitrogen-doped graphite exhibited reduced work function values (as low as 5.17 eV) at edge defect sites, which correlated with enhanced oxygen reduction reaction (ORR) activity. Although their study focused on ORR, the underlying mechanism, i.e., the facilitation of interfacial charge transfer through improved electron-donating ability, is also central to hydrogen evolution reactions. Thus, the agreement between our calculated work functions and experimental values reinforces the potential of C_4_N_2_ as an efficient metal-free catalyst.

We next examined whether C_36_N_18_ nanosheets could function as efficient electrocatalysts for HER activity. We investigated the optimal conditions for catalytic performance at three potential sites on C_36_N_18_: the hollow site at the center of a hexagon and the top site directly above each atom, as depicted in Figures 2 and S2.

**Figure 2:**
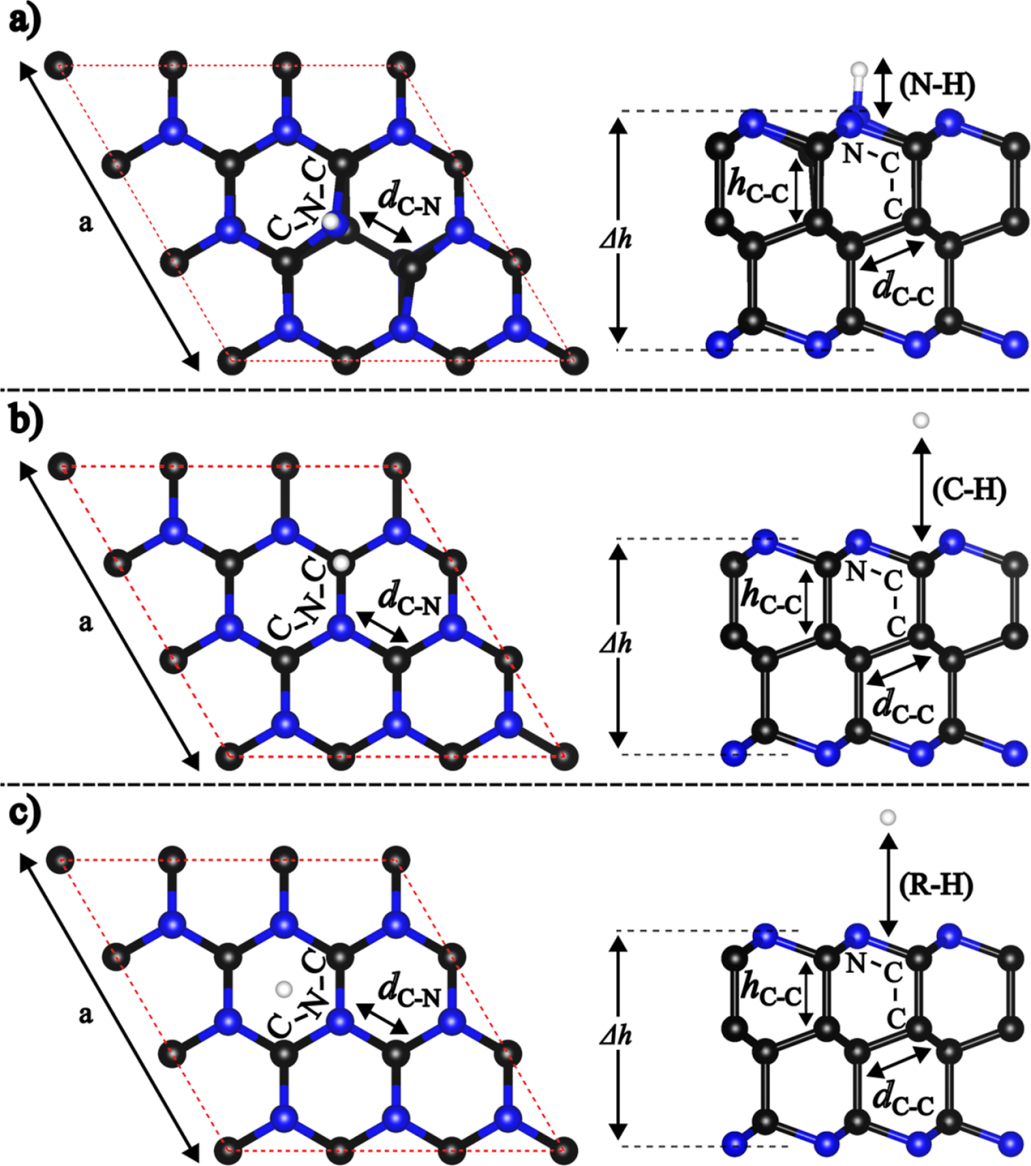
Optimized geometries of hydrogen adsorption sites on pristine AA′A^″^-C_36_N_18_, presented in top and side views. The investigated sites include the (a) nitrogen top, (b) carbon top, and (c) hollow sites. Bond distances and structural features upon adsorption are schematically shown and the respective values are in Table 1. The corresponding details for the ABC stacking configuration are provided in Figure S2 in the Supporting Information.

After structural optimization, the results show that, in the AA′A^″^ stacking configuration, the distances are 3.23 Å for both the carbon top and hollow sites and 1.03 Å for the nitrogen top. The ABC stacking configuration exhibits similar behavior, suggesting that different stacking arrangements impact marginally the hydrogen adsorption, as shown in Table 1. The interaction between H and C_36_N_18_ indicates physisorption at both the carbon top and hollow sites, as evidenced by the larger distances between H and the surface, with the lattice parameters, bond distances, and angles remaining essentially unchanged with H adsorption. In contrast, the interaction at the nitrogen top presents chemisorption characteristics, where a chemical bond forms between H and N, accompanied by structural changes near the adsorption site (see Figures 2 and S2, and Table 1).

The calculated Gibbs free energies (eq 3) for hydrogen adsorption on pristine C_36_N_18_ are 2.59, 2.59, and 2.23 eV for the AA′A^″^ stacking configuration, and 2.59, 2.59, and 2.28 eV for the ABC stacking, at the carbon top, hollow, and nitrogen top sites, respectively. It is well-established that platinum, an ideal catalyst for HER, has a Gibbs free energy close to zero, enabling efficient catalytic activity.^3^ On the other hand, the significantly positive Gibbs free energy values for pristine C_36_N_18_ indicate that hydrogen adsorption is less favorable, especially on the carbon top and hollow sites. However, stronger hydrogen adsorption was observed at the nitrogen top site, suggesting more promising interactions at that site when compared to the others.

Consequently, we analyzed the electronic band structure and projected density of states (PDOS) for all adsorption sites to gain insight into the hydrogen adsorption mechanism. First, we investigated the pristine C_36_N_18_ systems, as shown in Figures 3 and S3. Our calculations revealed a band gap of 4.41 eV for the AA′A^″^ stacking and 4.14 eV for the ABC stacking. The PDOS analysis indicates that the nitrogen p-orbitals dominate the VBM. In contrast, the CBM consists of a mixture of s- and p-orbitals from nitrogen and carbon atoms, consistent with previous studies.^14^

**Figure 3:**
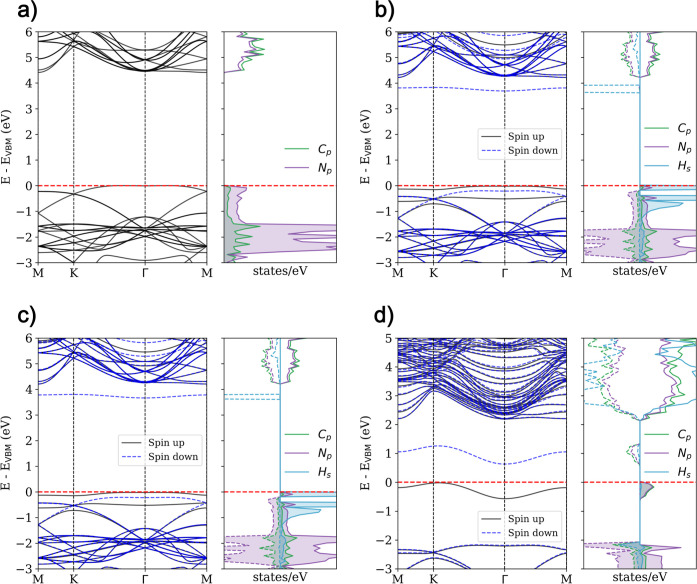
Electronic band structure and PDOS for (a) pristine AA′A^″^-C_36_N_18_ and H adsorbed on the (b) carbon top, (c) hollow, and (d) nitrogen top sites. Contributions from carbon, nitrogen, and hydrogen atoms to the valence and conduction bands are highlighted. The corresponding details for the ABC stacking configuration are provided in Figure S3 in the Supporting Information.

When hydrogen is adsorbed at the carbon top and hollow sites, the electronic structure is slightly perturbed, resulting in an electron trap state in the spin-down channel due to the H atom (see Figures 3 and S3). The PDOS around the Fermi level remains practically unaltered, indicating a weak interaction between hydrogen and the nanosheet, thus confirming the physisorption nature of the interaction at these sites.

In contrast, hydrogen adsorption at the nitrogen top site causes the bond to distort to accommodate nitrogen’s–3 oxidation state, involving one of the adjacent carbon atoms (see Figures 3 and S3). This interaction results in a new band in the spin-up and spin-down channels, with significant contributions from nitrogen and carbon p-orbitals. Moreover, all systems exhibit magnetic behavior after hydrogen adsorption, as evidenced by the PDOS asymmetry between the spin-up and spin-down bands. This asymmetry suggests the emergence of magnetism following hydrogen adsorption, particularly at the nitrogen top site.

Table 1 summarizes the calculated structural, electronic, and adsorption parameters related to HER. The similarities in bond distances, Gibbs free energy, electronic band structure, and PDOS imply that stacking configurations may have a marginal effect on enhancing catalytic activity. Furthermore, based on the Sabatier principle, a high positive Gibbs free energy makes adsorption less favorable, leading to the conclusion that pristine C_36_N_18_ is not well-suited for HER activity.

To enhance the catalytic efficiency of these nanosheets, we investigated substitutional doping with boron (B), silicon (Si), and phosphorus (P). Substitution was performed at a nitrogen site located on the top edge of the system, as this configuration is energetically favorable and allows a direct comparison between all dopants. In the AA'A″ and ABC-C_36_N_18_ nanosheets, surface nitrogen atoms are 3-fold coordinated with the carbon atoms, while the surface carbon atoms are additionally bonded to the atoms in the lower layer, making their removal structurally less favorable. This justifies the choice of the nitrogen site for substitution.

The B, Si, and P elements were selected based on their distinct electronic configurations, atomic sizes, and bonding characteristics, which are known to induce significant modifications in the local electronic structure of 2D materials. B, with its three valence electrons, has been widely reported to introduce acceptor states and promote favorable hydrogen adsorption by tuning the Fermi level and reducing the electronic bandgap in carbon-based catalysts. Si, as a group-IV element, introduces structural distortions and modifies orbital hybridization due to its larger atomic radius and lower electronegativity compared to nitrogen, potentially enhancing charge transfer and chemical reactivity. P, while isoelectronic with nitrogen, serves as a reference case that allows us to isolate the effects of local lattice deformation from purely electronic perturbations. This choice of dopants enables a systematic evaluation of how both electronic and geometric effects influence HER activity in doped systems. Furthermore, previous studies have shown improved HER activity in g-C_3_N_4_ doped with elements such as B, Si, and P^3,33^ (see Figure 4).

**Figure 4:**
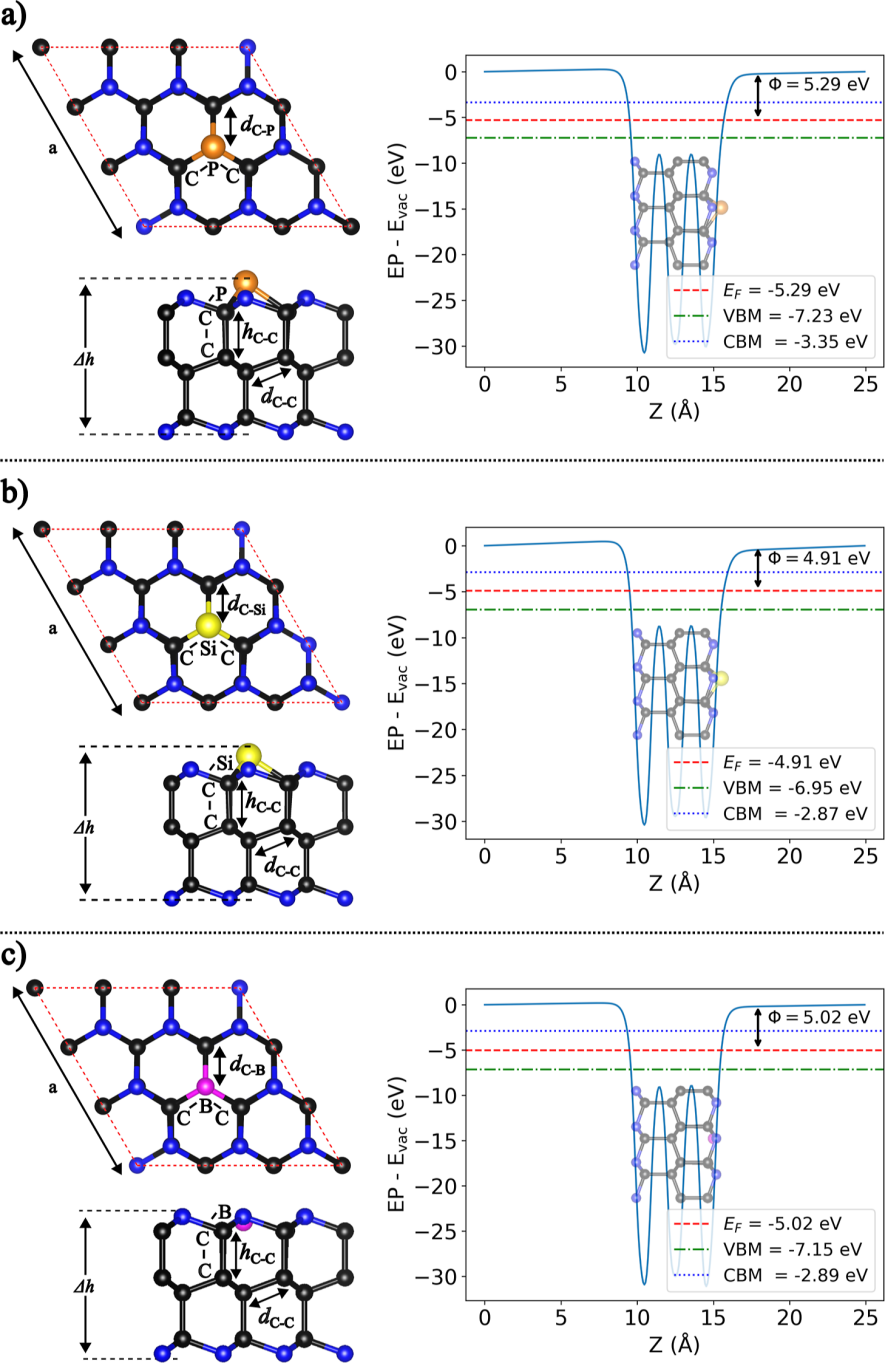
Schematic representation and work function (Φ) of optimized (a) P-doped, (b) Si-doped, and (c) B-doped AA′A^″^-C_36_N_17_ nanosheets, presented in top and side views. Structural parameters such as lattice constants, bond lengths, and bond angles are highlighted. The respective values are displayed in Table 2. The work function, Fermi energy, VBM, and CBM are referenced relative to the vacuum energy level. The corresponding details for the ABC stacking configuration are provided in Figure S4 in the Supporting Information.

Upon substituting nitrogen with dopants, optimization revealed that lattice parameters (*a*) and bond distances (*d*_C–C_ and *h*_C–C_) had only minor variations. In contrast, *d*_C‑X_ distances and C–C-X and C-X-C bond angles underwent significant changes, indicating that doping induced only localized structural perturbations (see Figures 4 and S4, and Table 2). The effects of P and Si substitutions on local structural parameters were similar, adjusting *d*_C‑X_ distances and altering the C–C-X and C–X–C bond angles to nearly 90° for C-X-C, reflecting an out-of-plane strain and an increase in Δ*h*. Here, Δ*h* refers to the vertical distance between the dopant and the nitrogen atom on the opposite edge, with P and Si positioned further from the surface than N. However, B-substitution produced a more planar C–X–C angle close to 120°, characteristic of sp^2^ hybridization, resulting in an almost flat local structure (see Table 2, Figures 4 and S4).

**Table 2:** Structural and Electronic Properties of Doped and Hydrogen-Adsorbed Doped C_36_N_17_: Lattice Parameter (*a*), Intralayer (*d*) and Interlayer (*h*) Distances, Thickness (Δ*h*), Intraplanar (C–X–C) and Interlayer (C–C–X) Bond Angles, Electronic Band Gap (*E*_g_), Work Function (Φ), and Gibbs Free Energies (Δ*G*), as Labeled in Figures 4 and 5 (AA′A^″^) and S4 and S5 in the Supporting Information (ABC). Distances are Given in Å, Energies in eV, Angles in Degrees, and X = P, Si, or B

system	*a*	bond length			Δ*h*	H distance	angle		band gap	Φ	Δ*G*
AA′A^″^		*d*_C–C_	*h*_C–C_	*d*_C‑X_			C–C-X	C-X-C			
P-doped	7.32	1.50	1.59	1.82	5.27		122.00	90.41	3.88	5.29	
H P-doped	7.32	1.50	1.59	1.82	5.27	3.16 (P–H)	122.00	90.41	3.86		2.62
Si-doped	7.33	1.51	1.58	1.82	5.21		119.45	92.86	4.08	4.91	
H Si-doped	7.33	1.51	1.58	1.81	5.19	1.46 (Si–H)	118.69	93.82	4.12		–1.51
B-doped	7.31	1.50	1.60	1.51	4.74		99.27	116.07	4.26	5.02	
H B-doped	7.31	1.50	1.61	1.58	4.87	1.20 (B–H)	111.75	105.58	3.89		–0.21
ABC											
P-doped	7.35	1.51	1.57	1.83	5.20		121.99	90.42	3.92	5.18	
H P-doped	7.35	1.51	1.57	1.83	5.20	3.14 (P–H)	121.99	90.42	3.89		2.58
Si-doped	7.37	1.51	1.57	1.82	5.15		119.35	92.99	4.00	4.99	
H Si-doped	7.37	1.51	1.56	1.81	5.12	1.46 (Si–H)	118.70	93.87	3.87		–1.50
B-doped	7.34	1.50	1.61	1.51	4.65		99.65	115.87	4.26	4.94	
H B-doped	7.34	1.50	1.58	1.58	4.78	1.20 (B–H)	111.81	105.64	3.78		–0.26

The effects of P and Si substitutions on local structural parameters were similar, adjusting *d*_C‑X_ distances and altering the C–C–X and C–X–C bond angles to nearly 90° for C–X–C, reflecting an out-of-plane strain and an increase in Δ*h*. Here, Δ*h* refers to the vertical distance between the dopant and the nitrogen atom on the opposite edge, with P and Si positioned further from the surface than N. However, B-substitution produced a more planar C–X–C angle close to 120°, characteristic of sp^2^ hybridization, resulting in an almost flat local structure (see Table 2, Figures 4 and S4).

Following the approach used for the pristine structure, we calculated Φ (eq 4) for all doped configurations. The calculated work functions for the doped C_36_N_17_ systems showed variations influenced by the type of dopant (see Table 2). For the P-doped system, the Φ value changed slightly, remaining within the range of noble metals, similar to the pristine case. On the other hand, the Si- and B-doped systems exhibited a reduction in Φ, with values around 5.00 eV. The relationship between Φ and catalytic performance in HER is complex and context-dependent. Therefore, the observed reduction in Φ for the Si- and B-doped systems must be understood in conjunction with other factors, such as electronic structure modifications and hydrogen adsorption behavior.^30,32^

We then modeled hydrogen adsorption directly on top of each dopant atom. These atoms are fully exposed on the surface and represent chemically distinct sites introduced by substitution. This configuration enables a controlled comparison of the dopants and aligns with our goal of evaluating the direct influence of the substituted atom on catalytic activity. Other nearby adsorption sites may also play a role, but were not explored in this work. In the final configuration, the H atom induced minor structural changes in the P- and Si-doped systems, while the B-doped case experienced a more notable shift, with bond angles transitioning from nearly sp^2^ to almost sp^3^ hybridization (see Table 2 and Figures 5 and S5). Notably, the H atom did not bond with the P atom, maintaining a P–H distance of approximately 3.15 Å, displaying physisorption behavior similar to that observed in the pristine case when H was adsorbed at the carbon top and hollow sites. Nevertheless, the interaction for the Si and B top sites resulted in a chemical bond between H and the dopant atoms, with H-dopant distances of 1.46 Å and 1.20 Å, respectively, indicating chemisorption. As for the pristine case, both stackings exhibited similar behavior, suggesting that different arrangements still do not significantly influence hydrogen adsorption in the doped systems.

**Figure 5:**
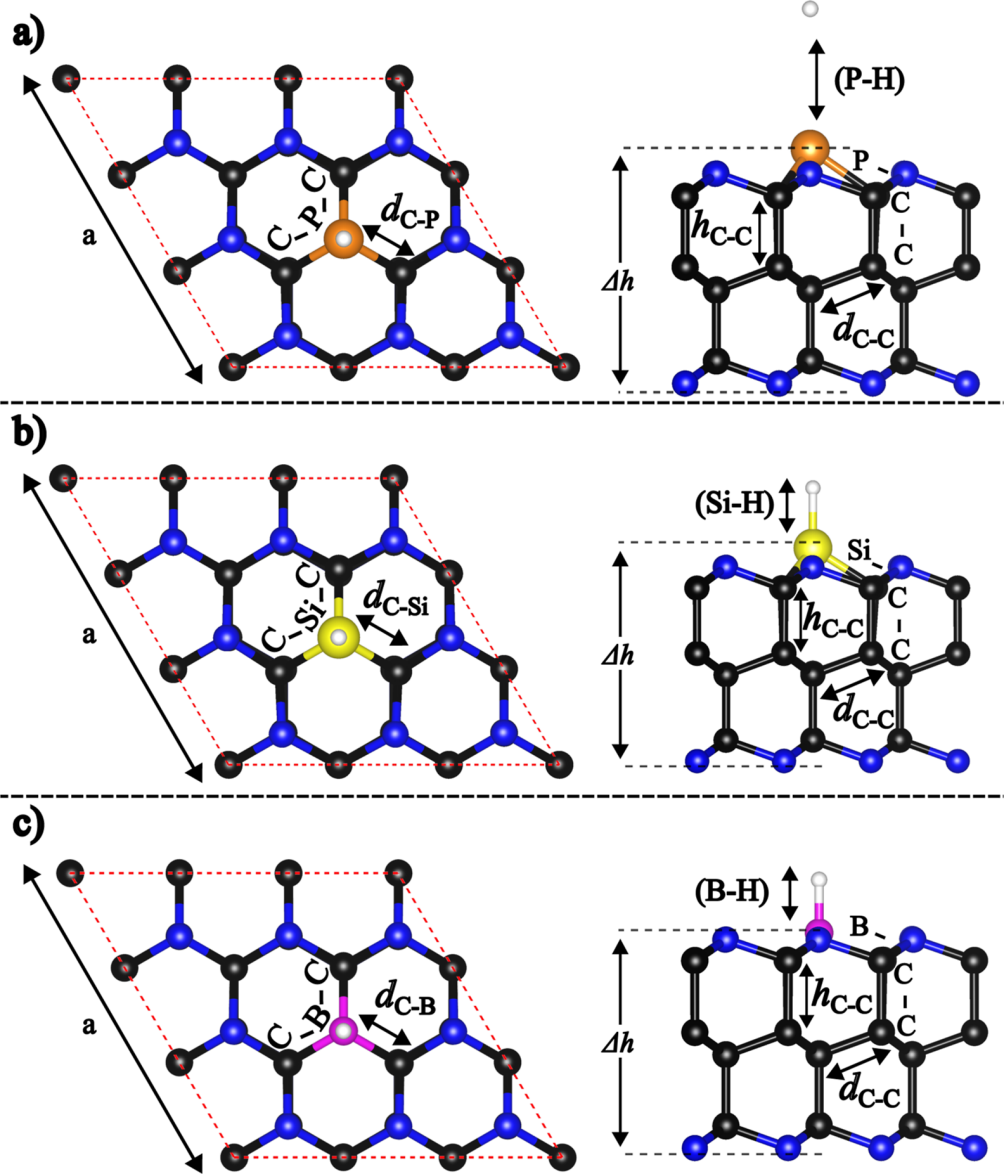
Optimized geometries of hydrogen adsorption sites on (a) P-doped, (b) Si-doped, and (c) B-doped AA′A^″^-C_36_N_17_, presented in top and side views. Changes in bond distances and structural features upon adsorption are shown. The respective values are in Table 2. The corresponding details for the ABC stacking configuration are provided in Figure S5 in the Supporting Information.

The calculated Gibbs free energies (eq 3) for H adsorption on the doped structures are summarized in Table 2. Notably, the B-doped system shows a marked improvement compared to pristine C_36_N_18_, with Gibbs free energy values approaching zero, indicative of efficient catalytic activity. Nevertheless, the P-doped system displays significantly positive Gibbs free energy values, while the Si-doped system exhibits highly negative values. This underscores the potential for enhanced catalytic activity through doping. To investigate the underlying reasons for these differences, we analyzed the electronic structure and PDOS of the doped systems before and after H adsorption.

Figures 6 and S6 present the band structures and PDOS before and after H adsorption, while Table 2 shows key properties. The P-doped case shows minimal changes in the band structure and PDOS character since P and N are isovalent. However, the PDOS for the P-doped system reveals contributions from p-orbitals due to P–C bonding, with a reduced band gap to approximately 3.90 eV. In the Si-doped case, the replacement of N by Si, which has one less valence electron than N, introduces notable changes. The main effect is the emergence of magnetism, as evidenced by the asymmetry between the PDOS for spin-up and spin-down electrons. This observation is in direct agreement with the findings reported by Laasonen et al.,^34^ who investigated over 6500 nitrogen-doped carbon nanotube (NCNT) configurations using machine learning. Their study demonstrated that a high degree of spin polarization at the adsorption site plays a particularly significant role in enhancing hydrogen adsorption strength. Additionally, a localized state appears in the spin-down channel, primarily due to the p-orbital of the Si atom. The band gap is reduced to around 4.00 eV if the localized state is not considered as effective narrowing of the gap. The B-doped system does not exhibit magnetism. With three valence electrons, B forms a localized state in the middle of the gap, primarily due to its p-orbitals. This results in a gap reduction to approximately 4.26 eV, similar to the Si case, where we consider this localized state not as an effective gap narrowing.

**Figure 6:**
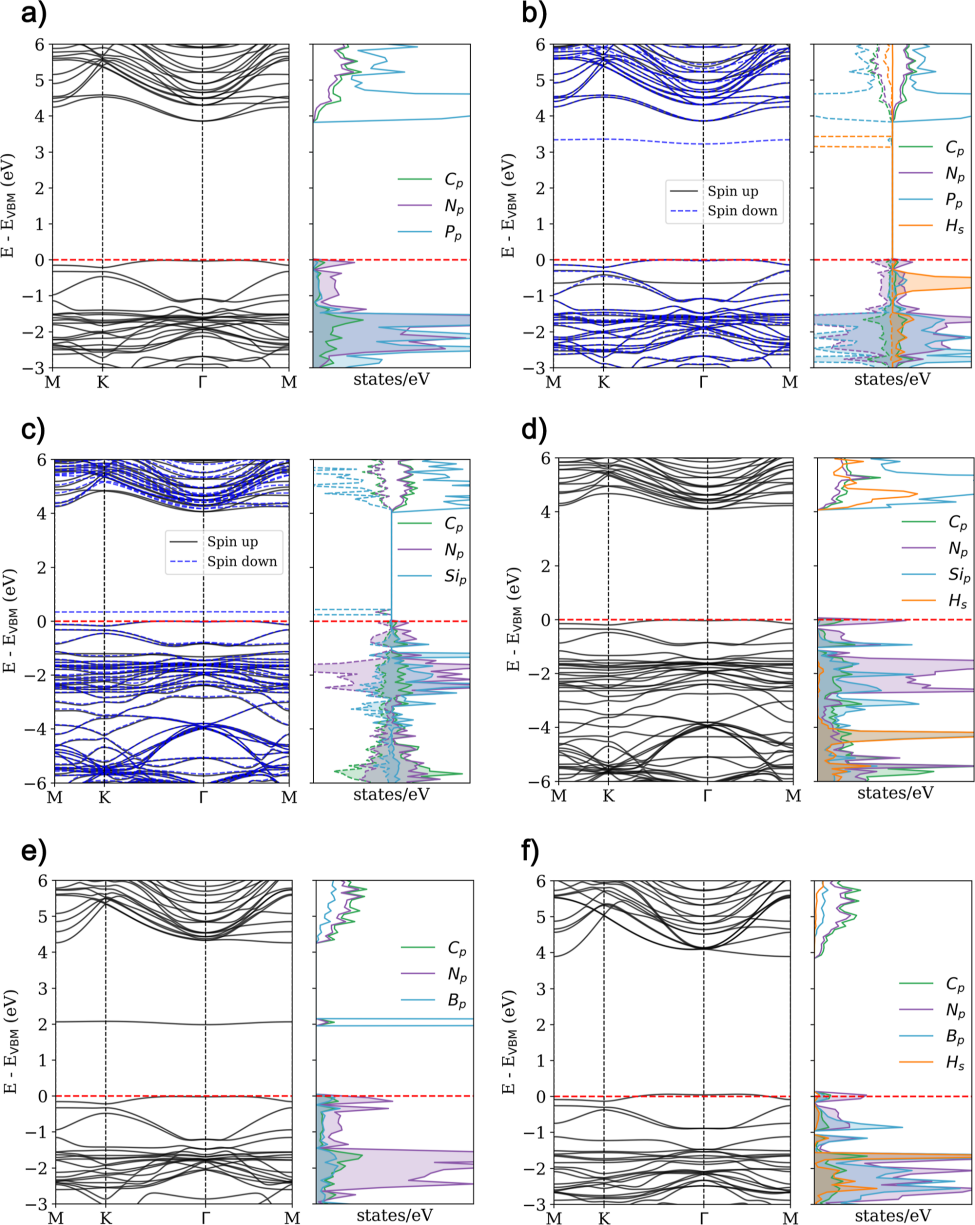
Electronic band structure and PDOS for (a) P-doped, (b) H adsorbed on P-doped, (c) Si-doped, (d) H adsorbed on Si-doped, and (e) B-doped, (f) H adsorbed on B-doped AA′A^″^-C_36_N_17_. Contributions from carbon, nitrogen, phosphorus, silicon, boron, and hydrogen atoms to the valence and conduction bands are highlighted. Details for the ABC stacking configuration can be found in Figure S6 in the Supporting Information.

After H adsorption, the band structure and PDOS changed, as shown in Figures 6 and S6. For the P-doped system, physisorption occurs, as evidenced by slight changes in the band structure and PDOS compared to the preadsorption state, along with two localized bands associated with the s-orbital of the H atom. Specifically, an s-orbital state in the spin-up channel is present within the valence band, and an s-orbital trap of H is in the spin-down channel close to the conduction band. This configuration introduces magnetism due to the unpaired electron of the H atom.

The Si- and B-doped systems, which initially exhibited trap states before H adsorption, undergo H chemisorption, with H atoms binding within these traps and effectively eliminating magnetism in both cases. For the Si-doped system, the band gap remains nearly unchanged, while a strong bond forms between H and Si. This is evidenced by the deeply bound s-orbitals of H observed in the PDOS, which likely contributes to the highly negative Δ*G*. In contrast, in the B-doped system, a partially filled band emerges at the top of the valence band, as revealed by the PDOS, which highlights hybridization between the H atom and nearby atoms. Similar partially filled bands have been observed in γ-graphyne after doping with B atoms, indicating a semiconductor-to-metal transition.^35^ This possible transition could explain the Δ*G* lower value in the B-doped system compared to that of the Si-doped case, emphasizing the role of dopant selection in optimizing catalytic performance. These observations support the interpretation that the dopant atoms themselves serve as the primary active sites for hydrogen adsorption. The localized trap states introduced by Si and B and their suppression or hybridization after H adsorption indicate direct electronic participation of the dopant atoms in the catalytic process.

To investigate the chemisorption interactions between H and both pristine and doped systems, focusing on cases where H binds to N, Si, or B atoms, we computed the charge density difference (see eq 5 and Figures 7 and S7) and Bader charge analyses. In the pristine C_36_N_18_, charge accumulation, highlighted in yellow, appears between the H and N atoms and near a C atom that distorts upon bonding to H. The Bader charge analysis reveals a charge transfer of 0.54 |*e*| from the H atom to C_36_N_18_, which is an indication of a polar covalent bond. For the Si- and B-doped systems, the charge distribution varies notably: in the Si-doped structure, the charge concentration centers around the H atom, while in the B-doped case, accumulation occurs beneath the B atom and between the B and neighboring C atoms. Here, Bader analysis indicates a charge transfer of 0.51 |*e*| from the H atom to the Si-doped C_36_N_17_ and 0.54 |*e*| to the B-doped C_36_N_17_, reflecting a shift in the interaction as compared to the pristine structure. These results suggest that Si and B dopants alter the electronic environment, enhancing the interaction with hydrogen and potentially boosting the catalytic efficiency.

**Figure 7:**
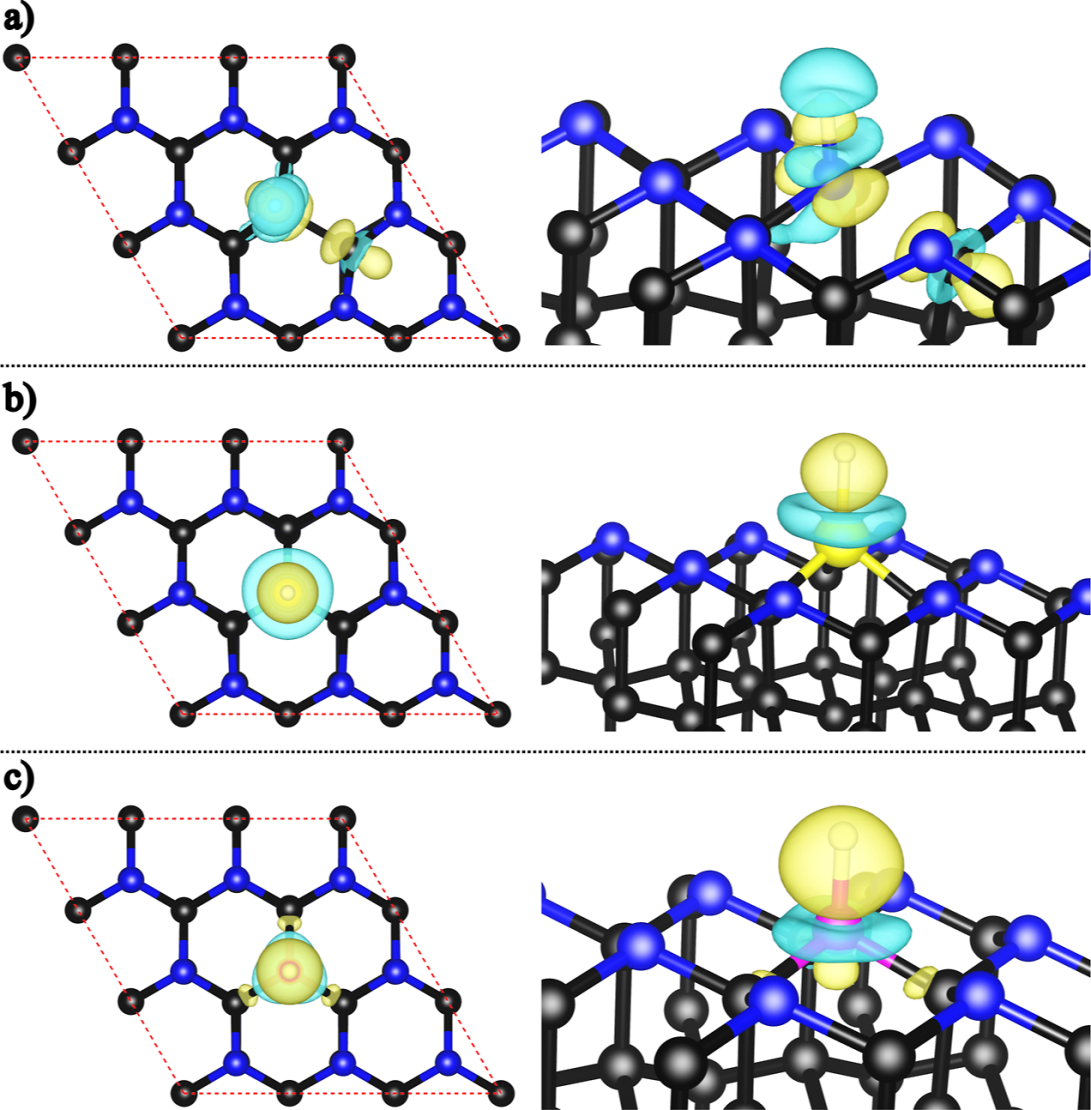
Charge density differences of hydrogen adsorption sites on (a) N-top of pristine (isosurface value 0.01 e/Bohr^3^), (b) Si-doped (isosurface value 0.0055 e/Bohr^3^), and (c) B-doped (isosurface value 0.012 e/Bohr^3^) AA′A^″^-C_36_N_17_, shown in top and side views. Charge accumulation (yellow) and depletion (cyan) regions are highlighted. Details for the ABC stacking configuration can be found in Figure S7 in the Supporting Information.

This distinct behavior can be attributed to the electronic characteristics of the dopants. Silicon, with a lower electronegativity and larger atomic radius than carbon and nitrogen, tends to localize the electron density around itself and the adsorbed hydrogen, forming a more polarized interaction. In contrast, boron, being more electronegative and possessing an empty p-orbital, can facilitate charge redistribution through its neighboring carbon atoms, leading to the observed accumulation beneath and around the B site. These differences in charge distribution reflect the way each dopant perturbs the local electronic structure and bonding characteristics of the C_36_N_17_ nanosheet.

Table 2 summarizes the structural, electronic, and adsorption parameters related to HER performance for doped systems. Stacking configurations show minimal impact on catalytic enhancement, as evidenced by the similarity in bond distances, Gibbs free energy, electronic band structure, and PDOS. Notably, despite having a slightly higher ϕ than the Si-doped system, the B-doped configuration exhibits the most favorable hydrogen adsorption free energy (Δ*G* ≈ −0.2 eV), indicating near-optimal catalytic behavior. These results support the notion that ϕ alone may not be sufficient to predict HER performance and must be considered in conjunction with other factors such as electronic structure modifications, charge transfer capability, and adsorption energetics. This interpretation is consistent with recent literature, which highlights that while ϕ relates to electron transfer, it is not a standalone predictor of HER activity and should be analyzed alongside Δ*G*, dopant-induced charge redistribution, and local electronic structure changes.^30^ In Figure S8 in the Supporting Information, we have also included the pH effects using the CHE (Computational Hydrogen Electrode) model.^36^ Our results, when accounting for pH effects, indicate that the B-doped site, identified as the most favorable for the hydrogen evolution reaction (HER), can exhibit enhanced catalytic performance. Specifically, the free energy of hydrogen adsorption (Δ*G*) at this site falls within the optimal range of −0.2 to 0.2 eV for pH values between 1 and 8.

Finally, achieving effective electrocatalysis typically requires metallic behavior to ensure efficient charge transfer. However, our results demonstrate that B-doped C_4_N_2_, despite being nonmetallic, exhibits a near-optimal Δ*G* for hydrogen adsorption, making it an excellent candidate as a cocatalyst. In this configuration, B-doped C_4_N_2_ would provide active sites for hydrogen adsorption, while a metallic material would supply electrons, ensuring efficient charge transfer. This design aligns with established strategies in electrocatalysis and photocatalysis, where nonmetallic cocatalysts enhance surface reactions while metallic components optimize electron transport.^37,38^ While our study focuses on the thermodynamic descriptor Δ*G*, we note that the geometric accessibility of the adsorption sites and the moderate adsorption energies suggest that kinetic barriers for H_2_ formation and desorption are unlikely to be prohibitive. In this context, and given the isolated nature of the adsorption sites in doped C_4_N_2_, the Volmer–Tafel mechanism is likely to dominate, where molecular hydrogen is formed by the recombination of two adsorbed H atoms.

## Conclusions

In conclusion, this study investigated the structural, electronic, and catalytic properties of pristine and doped C_4_N_2_ nanosheets as potential catalysts for the HER. Our results indicate that pristine C_36_N_18_ nanosheets exhibit limited HER activity due to their high positive Gibbs free energy. Doping with P maintains this limited activity, as the Gibbs free energy remains highly positive. In contrast, Si doping leads to highly negative Gibbs free energies, resulting in hydrogen adsorption that is too strong for effective HER catalysis. The most promising results were observed for B-doped C_36_N_17_ nanosheets, which exhibit a Gibbs free energy close to zero, indicating optimal hydrogen adsorption for efficient HER. Charge density and Bader charge analyses reveal substantial changes in the electronic environment upon doping. The modifications caused by B-doping significantly alter the electronic structure of C_36_N_18_, enhancing catalytic performance. Different stacking configurations (AA′A^″^ and ABC) have minimal impact on HER activity. Since B-doped C_4_N_2_ does not exhibit metallic behavior, it may not act as a primary electrocatalyst; however, its favorable Gibbs free energy suggests it can serve as an efficient cocatalyst, offering a promising strategy to improve overall catalytic performance.

## Supplementary Material



## Data Availability

Quantum ESPRESSO is a free, open-source code available at https://www.quantum-espresso.org/. The crystallographic information file (CIF) data for this article are available from Zenodo at 10.5281/zenodo.15489707.

## Notes

Structural models, adsorption geometries, electronic band structures with projected density of states (PDOS), work functions, and charge density difference plots for pristine and doped ABC-C_36_N_18_ nanosheets with and without hydrogen adsorption (PDF)
