# Supplementary material for: Metal-Free Doping Strategies in Two-Dimensional Carbon Nitride C4N2 for Enhanced Hydrogen Evolution Catalysis
Source: ACS Omega. 2025 Sep 16;10(38):44248–59. doi: 10.1021/acsomega.5c05773 (PMC12489677; doi:10.1021/acsomega.5c05773)
Supplement: Supplementary file 1 [file ao5c05773_si_001.pdf]

Support Information: Metal-Free Doping Strategies in Two-Dimensional Carbon Nitride C_4N_2 for Enhanced Hydrogen Evolution Catalysis

Bruno Ipaves,[*,†] João F. Justo,[‡] James M. de Almeida,[¶] Lucy V. C. Assali,[§] and Pedro Alves da Silva Autreto[*,†]

†*Center of Natural and Human Sciences, Federal University of ABC (UFABC), Santo André, 09280-560, São Paulo, Brazil*

‡*Escola Politécnica, University of São Paulo (USP), São Paulo, 05508-010, São Paulo, Brazil*

¶*Ilum School of Science, Brazilian Center for Research in Energy and Materials (CNPEM), Campinas, 13083-970, São Paulo, Brazil*

§*Institute of Physics, University of São Paulo (USP), São Paulo, 05508-090, São Paulo, Brazi*

E-mail: ipaves.bruno@ufabc.edu.br; pedro.autreto@ufabc.edu.br

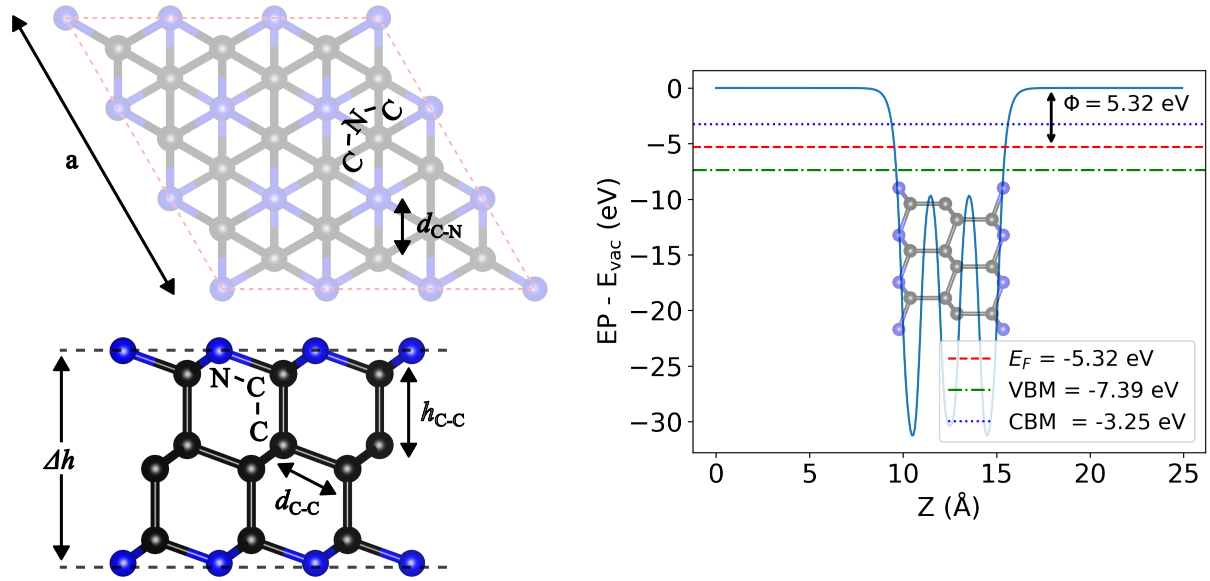

Figure S1: Schematic representation of the optimized pristine ABC-stacked $C_{36}N_{18}$ nanosheet, shown in top and side views (left), along with its calculated work function (Φ). Key structural parameters, lattice constants, bond lengths, and bond angles are highlighted, with numerical values provided in Table 1 of the main text. The work function, Fermi energy, valence band maximum (VBM), and conduction band minimum (CBM) are referenced relative to the vacuum energy level.

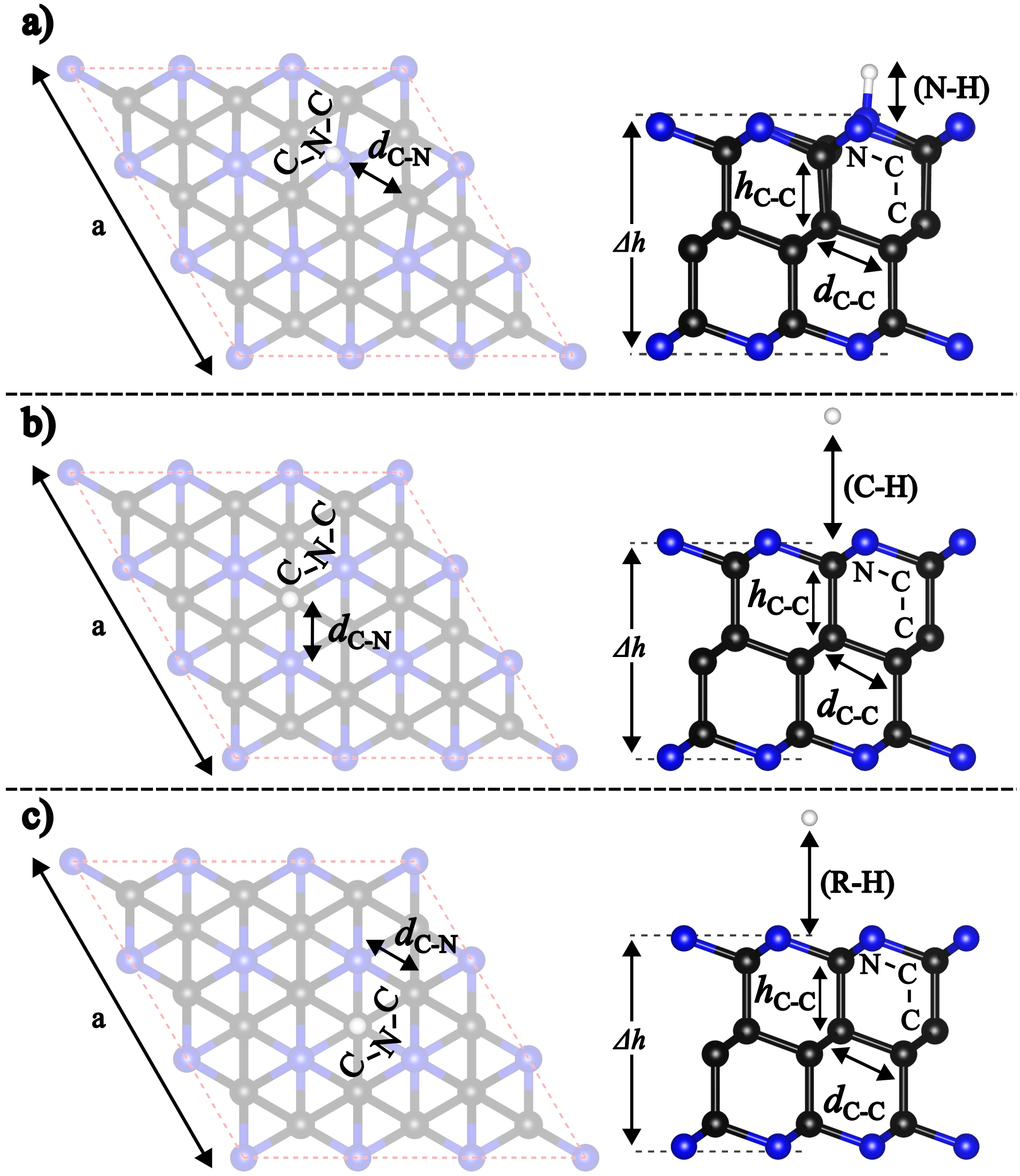

Figure S2: Optimized geometries of hydrogen adsorption on the pristine ABC-stacked $C_{36}N_{18}$ nanosheet, shown in top and side views. The investigated adsorption sites are: (a) nitrogen top, (b) carbon top, and (c) hollow. Structural modifications upon H adsorption, including changes in bond lengths and local geometry, are highlighted. The corresponding numerical values are reported in Table 1 of the main text.

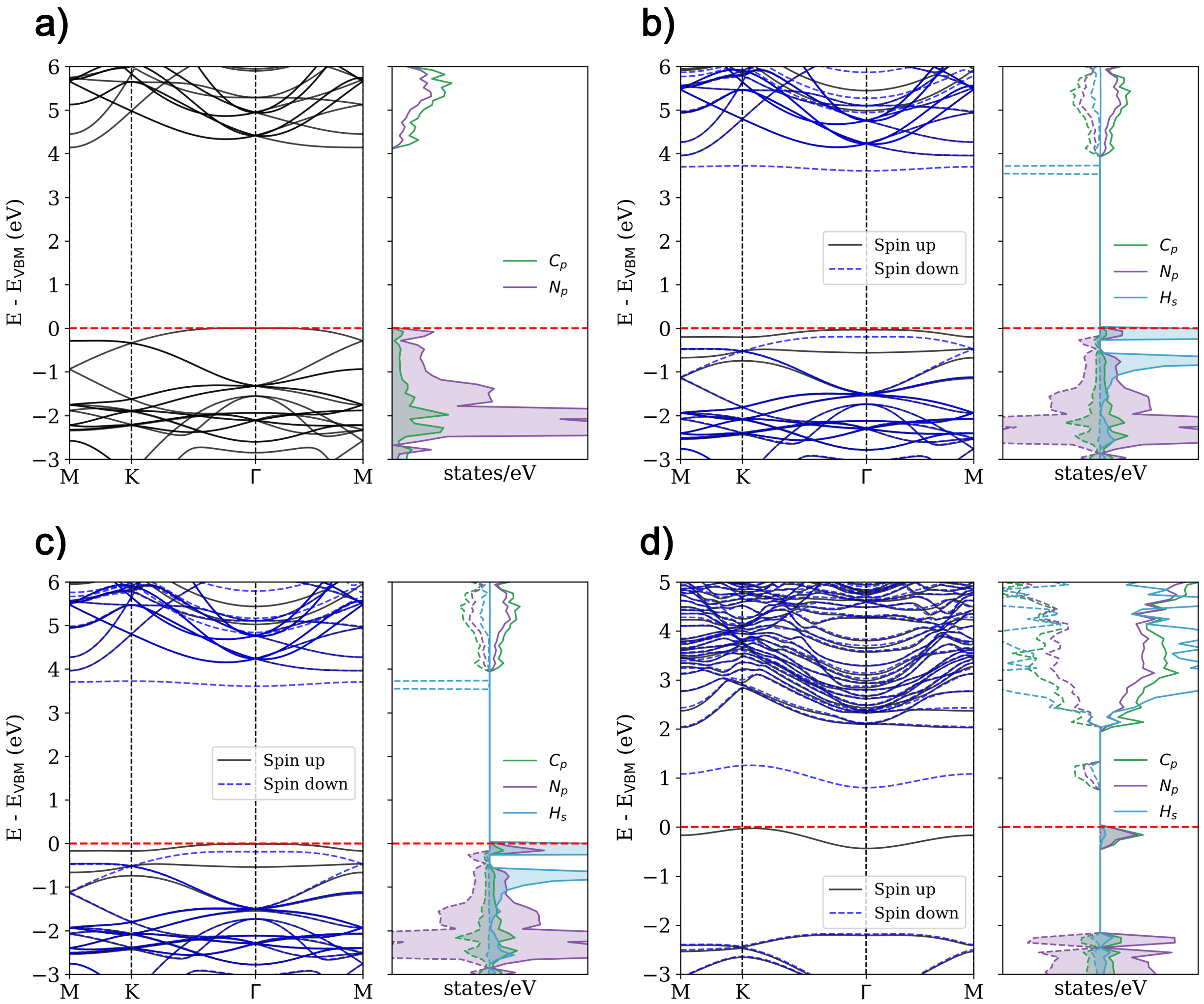

Figure S3: Electronic band structures and projected density of states (PDOS) for the ABC-stacked $C_{36}N_{18}$ nanosheet, as discussed in the main text: (a) pristine structure, and with H adsorbed (b) on top of a carbon atom, (c) at the hollow site, and (d) on top of a nitrogen atom. Contributions from carbon, nitrogen, and hydrogen atoms to the valence and conduction bands are highlighted.

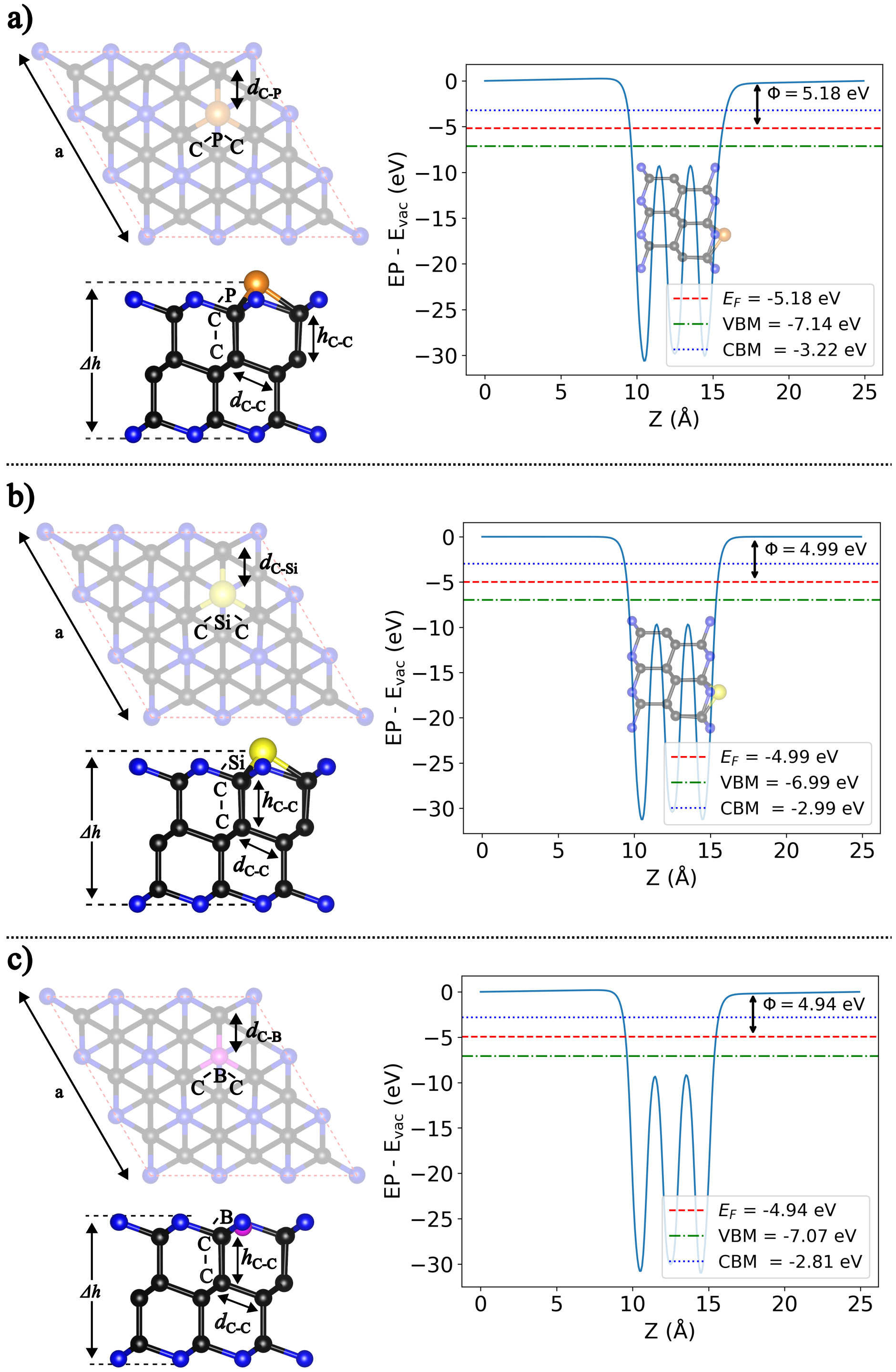

Figure S4: Schematic representation of the optimized (a) P-doped, (b) Si-doped, and (c) B-doped ABC-stacked $C_{36}N_{17}$ nanosheets, shown in top and side views, along with their respective calculated work functions (Φ). Key structural parameters (lattice constants, bond lengths, and bond angles) are highlighted, with numerical values provided in Table 2 of the main text. The work function, Fermi energy, valence band maximum (VBM), and conduction band minimum (CBM) are referenced relative to the vacuum energy level.

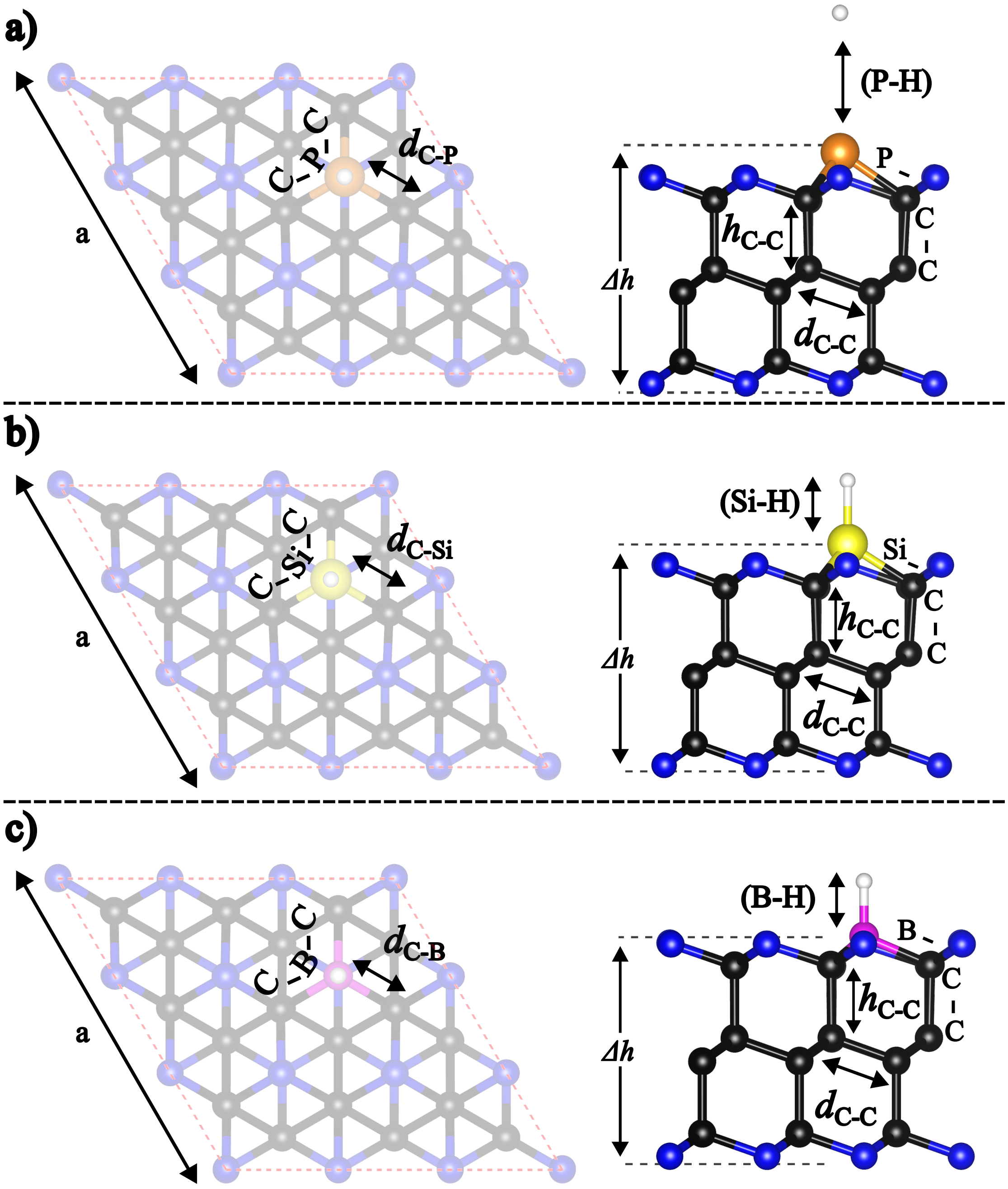

Figure S5: Optimized geometries of hydrogen adsorption on (a) P-doped, (b) Si-doped and (c) B-doped ABC-stacked $C_{36}N_{17}$ nanosheets, shown in top and side views. Structural changes upon adsorption, including variations in the bond lengths and local geometry, are highlighted. The corresponding numerical values are provided in Table 2 of the main text.

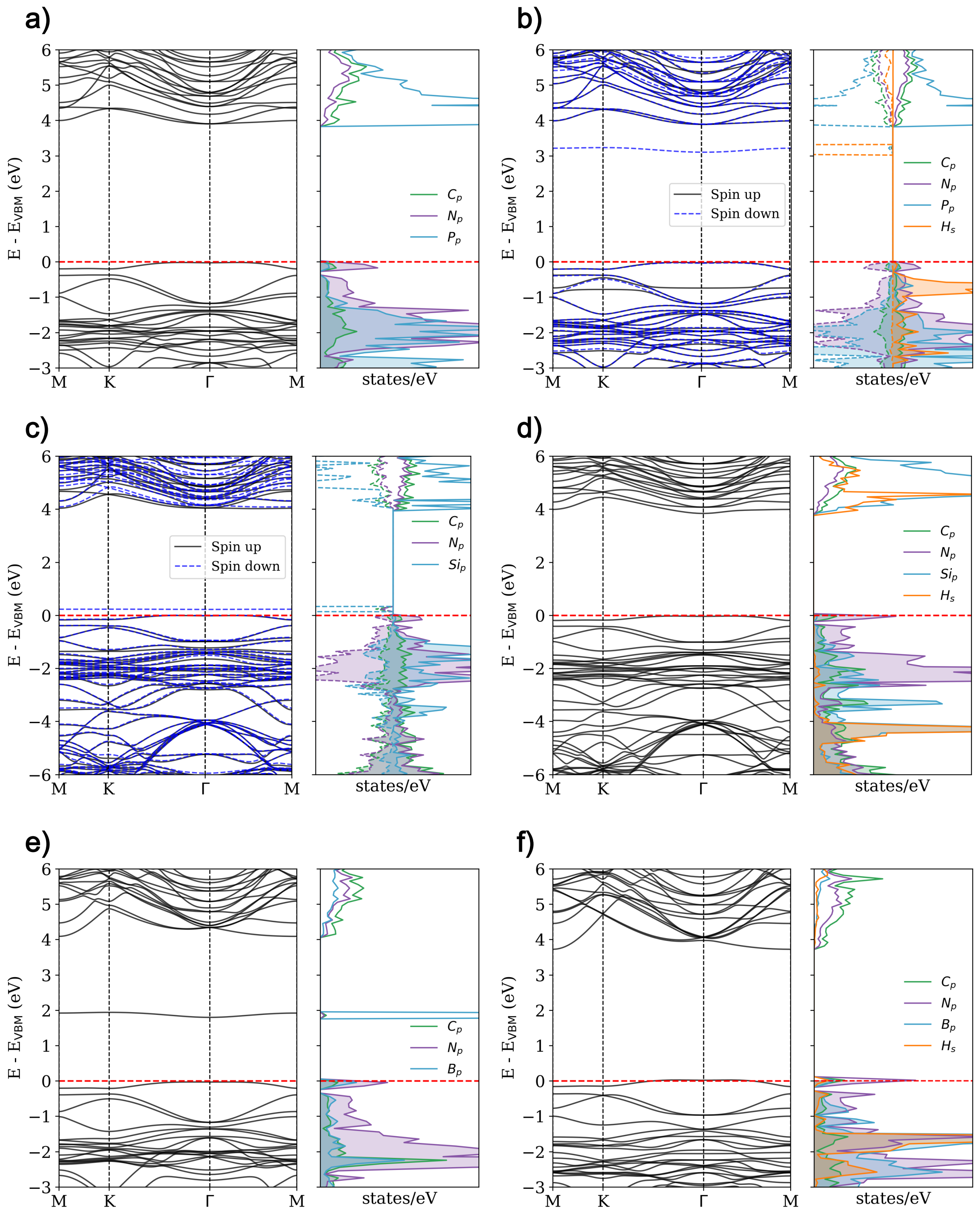

Figure S6: Electronic band structures and projected density of states (PDOS) for the doped ABC-stacked $C_{36}N_{17}$ nanosheet, as discussed in the main text: (a) P-doped, (b) with H adsorbed on the P-doped structure, (c) Si-doped, (d) with H adsorbed on the Si-doped structure, (e) B-doped, and (f) with H adsorbed on the B-doped structure. The contributions from carbon, nitrogen, phosphorus, silicon, boron, and hydrogen atoms to the valence and conduction bands are highlighted.

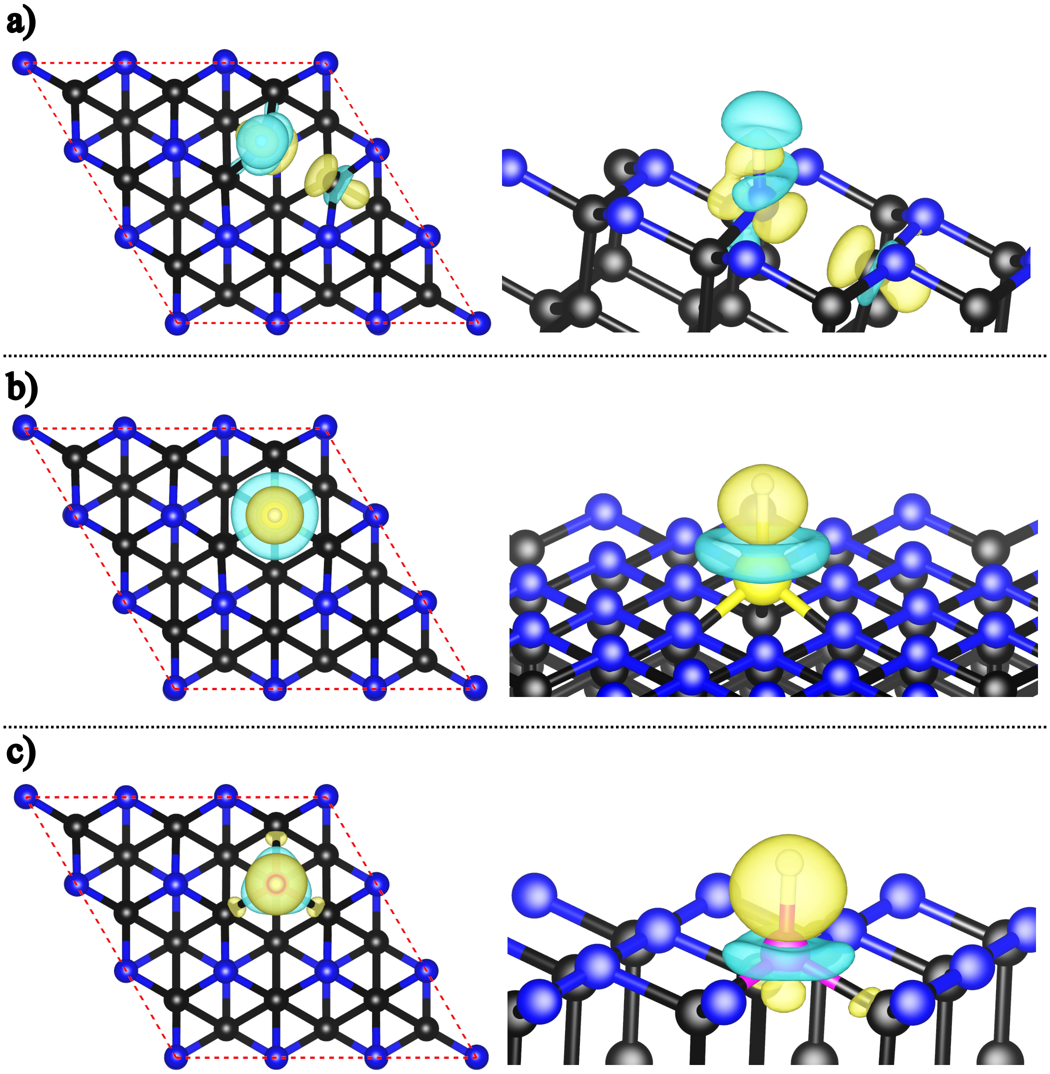

Figure S7: Charge density differences of hydrogen adsorption sites on the ABC-stacked $C_{36}N_{17}$ nanosheet, as discussed in the main text: (a) N-top site of the pristine structure (isosurface value 0.01 e/Bohr^3), (b) Si-doped structure (0.0055 e/Bohr^3), and (c) B-doped structure (0.012 e/Bohr^3), shown in top and side views. Charge accumulation (yellow) and depletion (cyan) regions are highlighted.

We have now applied the standard solvation model as an initial approach to address this aspect in our system.[1]

$$\Delta G = \Delta G_{H*} + k_B.T.pH.ln(10) \tag{1}$$

where ΔG_{H*} was the value previously calculated, k_B is the Boltzmann constant, pH is the pH value.

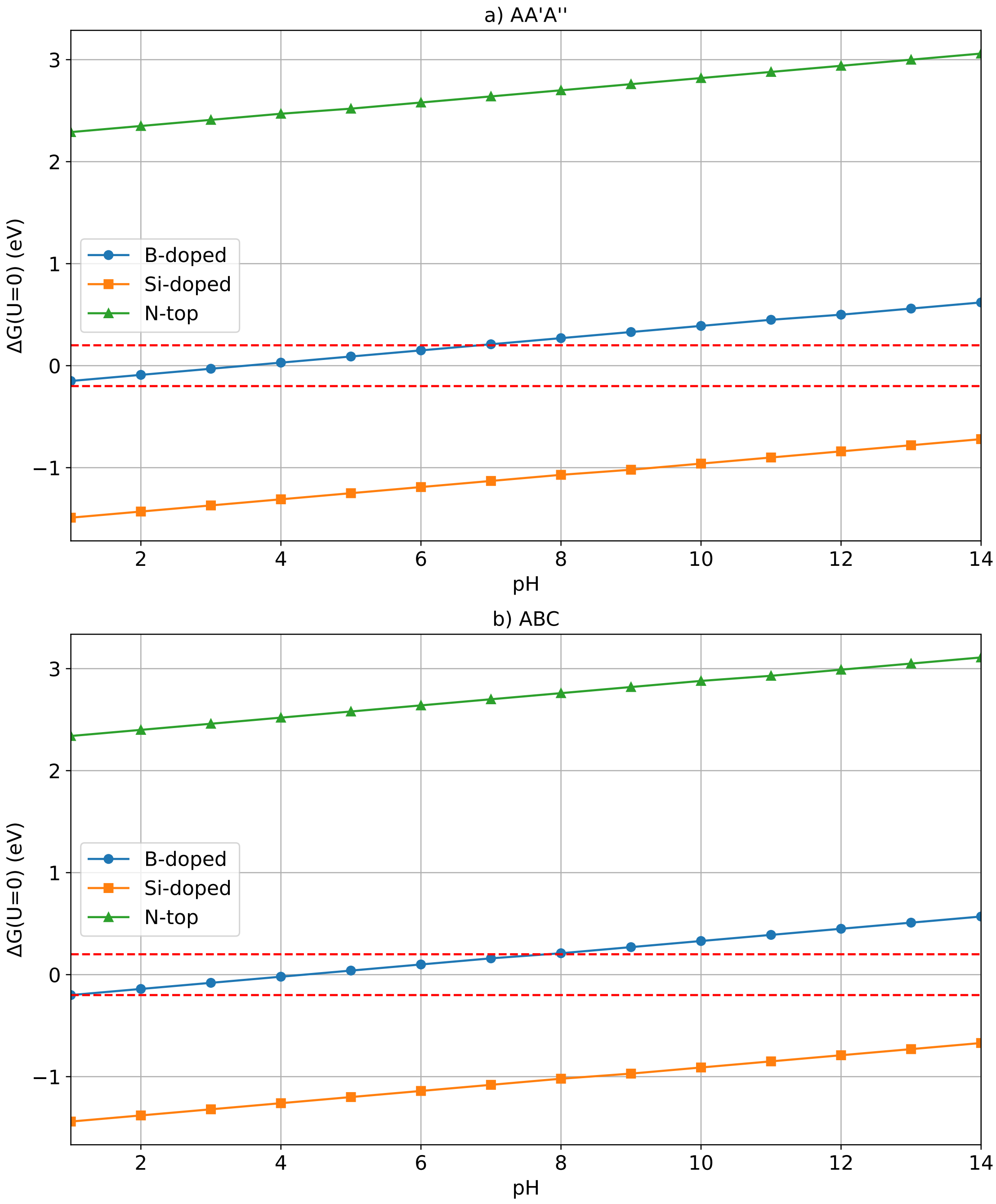

Figure S8: Free energy variation as a function of pH at U = 0 V vs RHE for (a) AA′A″ stacking and (b) ABC stacking. Red dashed lines indicate the optimal region for HER.

References

(1) Norskov, J. K.; Rossmeisl, J.; Logadottir, A.; Lindqvist, L.; Kitchin, J. R.; Bligaard, T.; Jonsson, H. Origin of the overpotential for oxygen reduction at a fuel-cell cathode. *The Journal of Physical Chemistry B* **2004**, *108*, 17886–17892.
